# High-precision automatic identification method for dicentric chromosome images using two-stage convolutional neural network

**DOI:** 10.1038/s41598-023-28456-9

**Published:** 2023-02-06

**Authors:** Xiang Shen, Tengfei Ma, Chaowen Li, Zhanbo Wen, Jinlin Zheng, Zhenggan Zhou

**Affiliations:** 1 School of Mechanical Engineering and Automation, Beihang University, Beijing, 100083 China; 2 Beijing Huironghe Technology Co., Ltd., Beijing, 101102 China; 3 Ningbo Institute of Technology, Beihang University, Ningbo, 315800 China

**Keywords:** Cellular imaging, Chromosomes, Cytogenetics, Computer science

## Abstract

Dicentric chromosome analysis is the gold standard for biological dose assessment. To enhance the efficiency of biological dose assessment in large-scale radiation catastrophes, automatic identification of dicentric chromosome images is a promising and objective method. In this paper, an automatic identification method for dicentric chromosome images using two-stage convolutional neural network is proposed based on Giemsa-stained automatic microscopic imaging. To automatically segment the adhesive chromosome masses, a *k*-means based adaptive image segmentation and watershed segmentation algorithm is applied. The first-stage CNN is used to identify the dicentric chromosome images from all the images and the second-stage CNN works to specifically identify the dicentric chromosome images. This two-stage CNN identification method can effectively detects chromosome images with concealed centromeres, poorly expanded and long-armed entangled chromosomes, and tricentric chromosomes. The novel two-stage CNN method has a chromosome identification accuracy of 99.4%, a sensitivity of 85.8% sensitivity, and a specificity of 99.6%, effectively reducing the false positive rate of dicentric chromosome. The analysis speed of this automatic identification method can be 20 times quicker than manual detection, providing a valuable reference for other image identification situations with small target rates.

## Introduction

**Figure 1:**
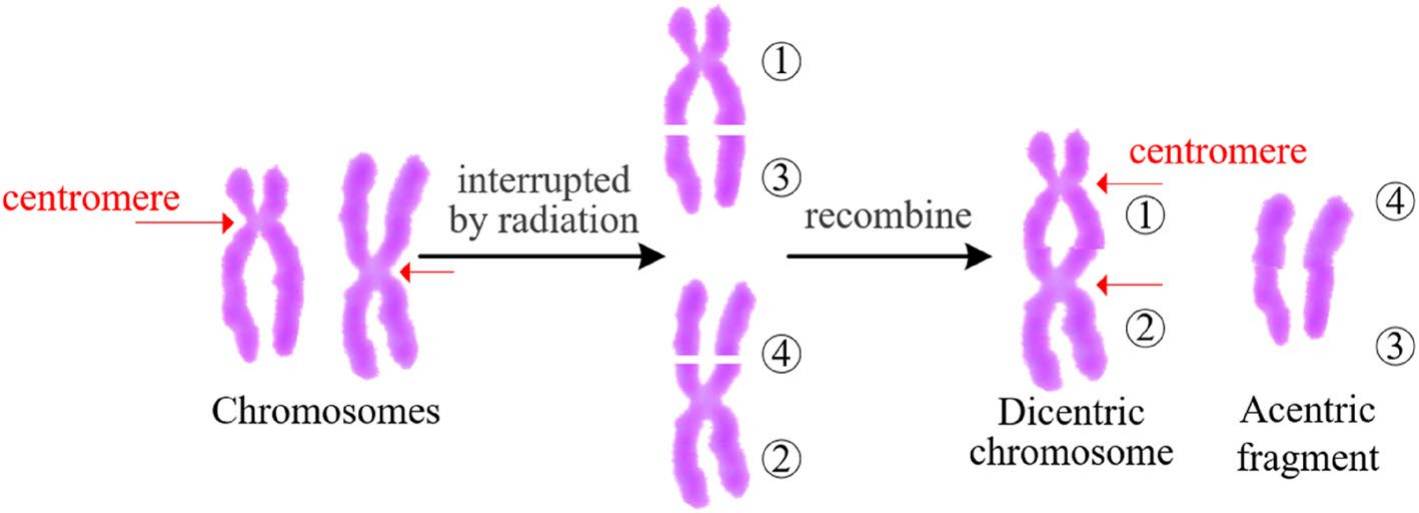
Generation process of DIC.

In time biological dose assessment is crucial for radiation disaster rescue^1^. Dicentric chromosome analysis is considered as a gold standard^2^ for biological dose assessment by the International Atomic Energy Agency. Figure 1 shows a typical generation process of a dicentric chromosome (DIC). When two broken chromosomes are close enough, they may recombine together and generate a DIC, while the two remaining segments without centromeres may recombine and generate an acentric fragment. The generation of a DIC is accompanied with the generation of an acentric fragment. Since manual identification for DIC is time-consuming and sometimes subjective, automatic identification systems have great application prospect to improve the biological dose assessment in large-scale radiation catastrophes^3,4^.

Conventional methods for dicentric chromosome identification firstly extracts the medial axis of the chromosome image and then identifies the centromeres based on the morphological properties of the chromosome. The commonly used medial axis extraction methods rely on chromosome cross-section pixel information^5^ and various curve fitting methods^6–8^, while some medial axis extraction methods implement handwritten word recognition^9^, skeleton pruning techniques^10,11^ and iterative skeletonisation technique^12^. For the identification of the centromere, the centromere positions can be calculated by convex and concave property^10,12^, and the pixel information of the chromosomes^6,7,13^. The centromere can also be detected by applying multicolor fluorescence in situ hybridization images^14–17^. Since the morphology of chromosomes is diverse, the conventional methods cannot easily identify different morphologies of chromosomes, limiting the identification accuracy.

To enhance the identification accuracy for DIC images, in recent years, an increasing number of researchers have employed deep learning algorithms for identification and classification of cell images, such as binucleated lymphocytes images^18^, human fundus images^19^, skin cancer images^20^, and ovary images^21^. Machine learning methods such as the Feature Pyramid Network^22^, support vector machine^23^, hierarchical multilayer neural network with error back propagation training algorithm^24^, and Siamese network^25^ have been utilized in the automatic identification of DIC images. Some methods have been implemented to improve the identification accuracy, such as filtering out the undesirable images of metaphase cells by the image filtering neural network^22^ or image morphological features^26,27^, straightening chromosomes before identification, and manual proofreading only for DICs after automatic identification^28^.

Since DIC only takes a small proportion of chromosomes in metaphase cell, many automatic identification algorithms have a high false-positive rate of DIC, especially for low-dose irradiated samples^29,30^. In this paper, a high-precision automatic identification algorithm for DIC images using two-stage convolutional neural network (CNN) is proposed. A self-developed automatic microscopy is adopted to acquire Giemsa-stained metaphase cell images. *k*-means based adaptive image segmentation and watershed segmentation algorithms are used to extract individual chromosomes and automatically segmentthe adhesive chromosome masses. The first-stage CNN is used to identify the dicentric chromosome images from all chromosome images and the second-stage CNN works to specifically identify the dicentric chromosome images. This method can provide a valuable reference for other image identification situations with small target rates.

## Sample preparation and image acquisition

### Sample preparation

40 slides of chromosome samples are provided by Henan Institute for Prevention and Treatment of Occupational Disease, China from 20 healthy individuals (10 female and 10 male) in the range of 25 to 40 years old. Each volunteer provides 5 ml of peripheral blood. All the volunteers are nonsmokers in good health condition (no visible infections such as flu or pneumonia) during blood collection, and have not received any medical exposure in the previous year. To obtain DICs, all blood samples are irradiated by gamma radiation of 2 Gy.

All experimental protocols in this paper were approved by the Ethic Committee of the Henan Institute for Prevention and Treatment of Occupational Disease, China, conformed to the Declaration of Helsinki, including:

- Collection of human blood.
- Cell culture and slide production.
- Collection of microscopic cell images.
- Automatic identification and manual analysis of metaphase cell images.

Slides of the chromosome samples are prepared according to the guidelines and regulations issued by the International Atomic Energy Agency^2^. Informed consent was obtained from all subjects and/or their legal guardian(s).

### Image acquisition system

**Figure 2:**
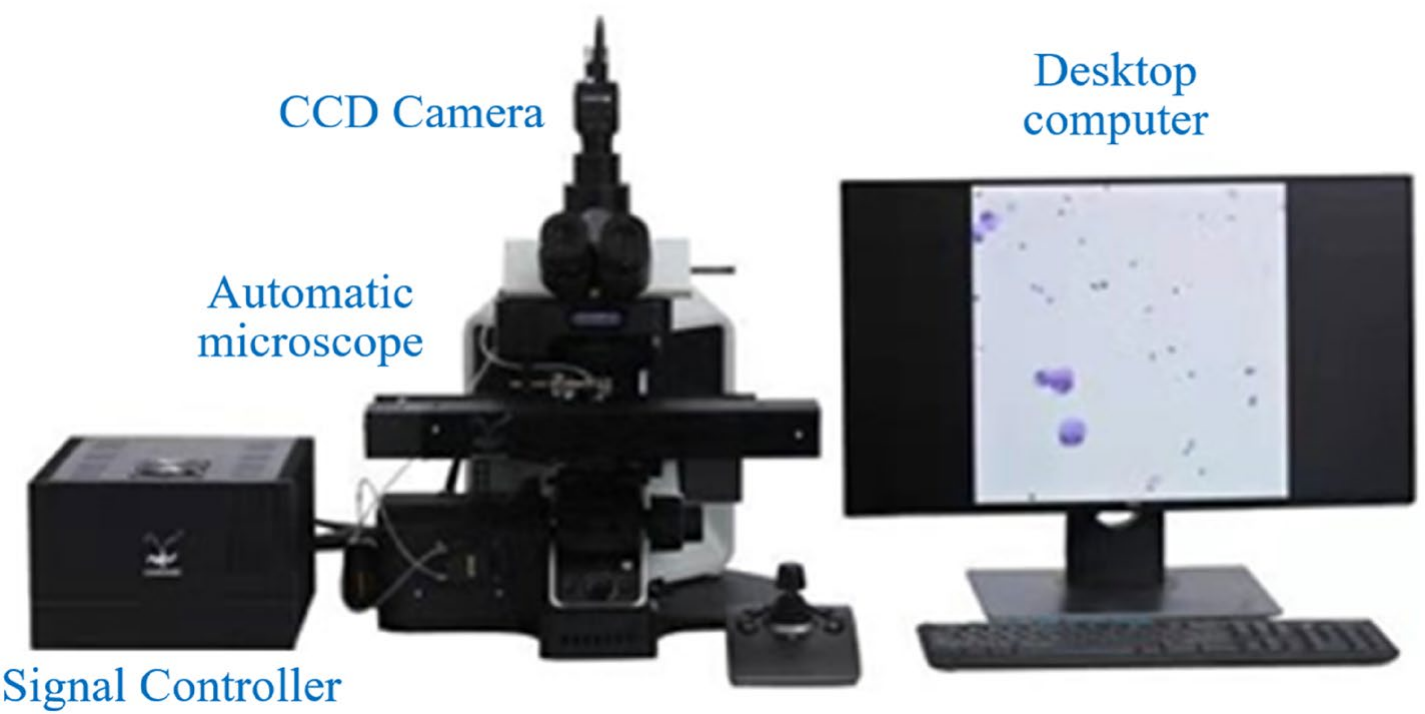
Automatic chromosome imaging system.

As shown in Fig. 2, the automatic chromosome imaging system consists of: (1) Desktop with a 16-core Intel Core i9-12900K CPU (64G memory) and NVIDIA GeForce GTX 3090 GPU (24G memory); (2) CCD camera (Lumenera, Canada); (3) Automatic microscope with motion control stage, focal length adjustment device and lens switching device; (4) Signal controller for the motion control platform.

Since the metaphase cell images need to be captured into 63 × or 100 × objective, the slides are initially autofocused and the images are taken at 10 × objective to speed up the imaging process. The metaphase cells are detected in the obtained images using the automatic location algorithm to be introduced later. The relative coordinates of the metaphase cell in the image and the location of the image on the slide are combined to compute the location of the metaphase cell on the slide. The metaphase cells are then automatically focused and its images are captured by the automatic scanning stage where the microscope is switched to 100 × objective.

## Automatic identification method using two-stage convolutional neural network

The automatic image identification method includes four steps. First, in the images at 10 × objective, the location of the metaphase cells is captured to assist the automatic microscope to acquire the metaphase cell images at 100 × objective. Then, chromosome images are extracted from the metaphase cell image. After that, the adhesive chromosome masses are segmented into individual chromosomes. Finally, the dicentric chromosomes are detect and identified by the two-stage CNN.

### Automatic metaphase cell locating algorithm

**Figure 3:**
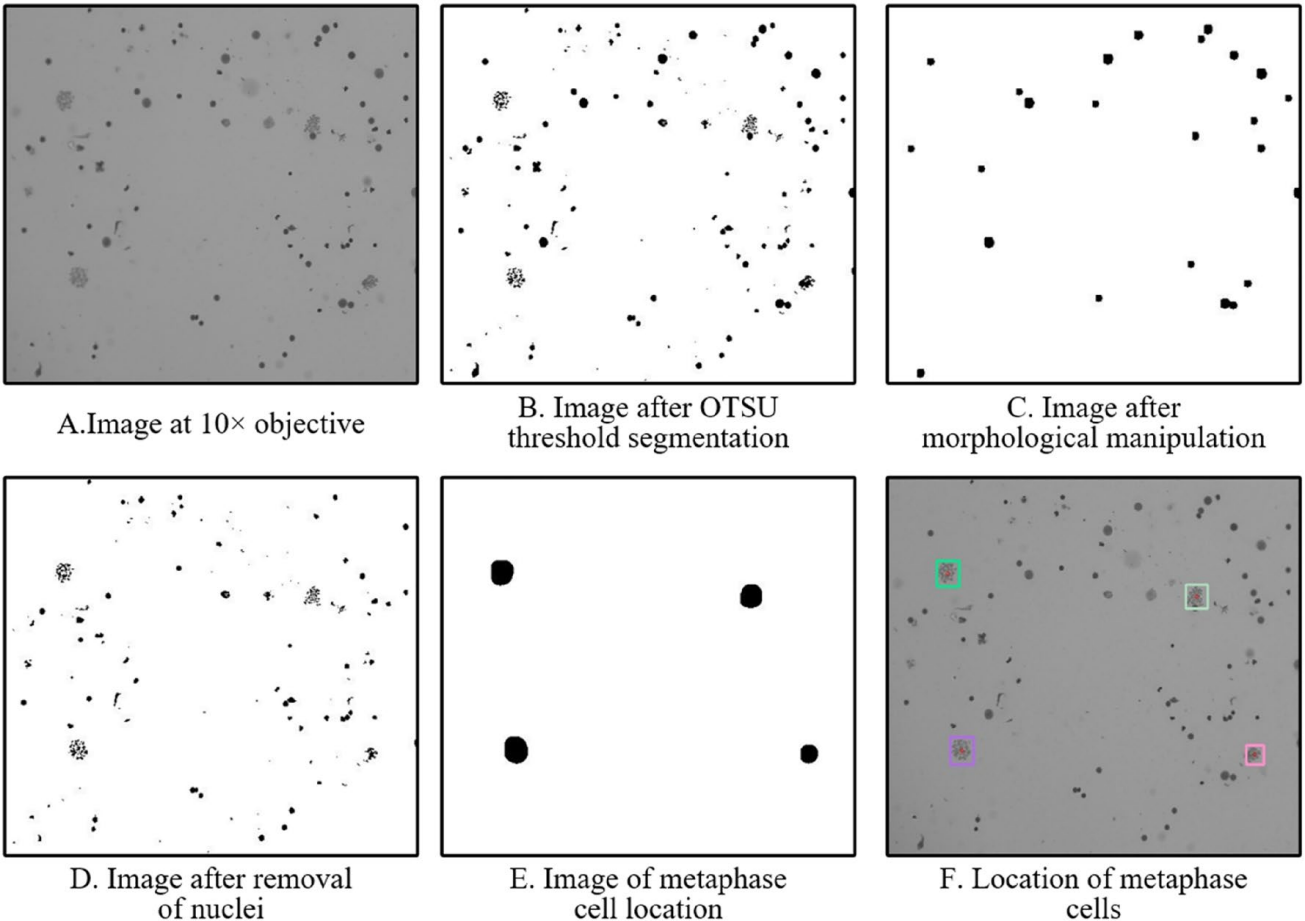
Process of the automatic metaphase cell locating algorithm.

As shown in Fig. 3A, at 10 × objective, the metaphase cells can be observed together with many impurities and nuclei not in the intermediate division stage. The morphological manipulation is used to distinguish them since the metaphase cells contain a significant number of slim chromosomes and seem “pore-like”, whereas nuclei and impurities appear “blocky-like”. The OTSU threshold segmentation^31^ is first conducted and Fig. 3B is obtained. Then Fig. 3B is subjected to the morphological erosion and expansion procedures to eliminate the metaphase cells. The result is shown in Fig. 3C. The big nuclei and impurities are removed by subtracting the contents of Fig. 3C from Fig. 3B, as shown in Fig. 3D. Figure 3D is further morphologically inflated, followed by erosion to remove tiny bits of impurities. Figure 3E is created by morphologically inflating the image to highlight the location of metaphase cells. Finally, the centroids and location of all metaphase cells can be obtained, as Fig. 3F shows. This assists the automatic microscope to focus on the metaphase cell and scan the images at 100 × objective. The threshold of chromosome number for the screening of metaphase cell images is set to 46 ± 3 due to the presence of adherent chromosome clusters and acentric fragments.

### Chromosome extraction algorithm

**Figure 4:**
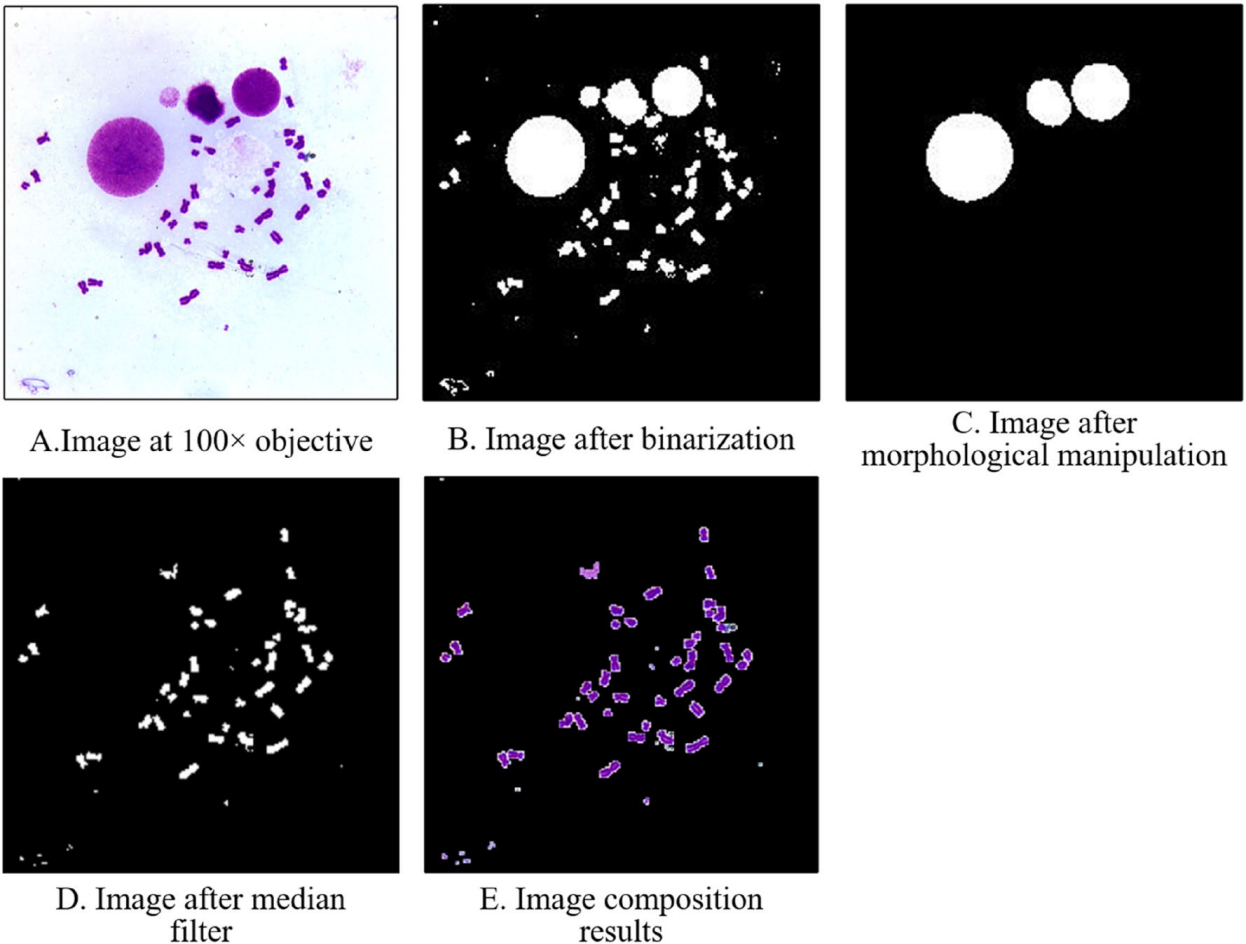
Process of the chromosome extraction algorithm.

Figure 4A shows the metaphase cell images obtained by the automatic microscope at 100 × objective. The metaphase cell images are first clustered using the *k*-means clustering algorithm (*k* = 2), and then binarized based on the clustering results and obtain Fig. 4B. Since some nuclei and contaminants always occur around the metaphase cells, morphological erosion and expansion procedures are conducted for Fig. 4B, C is obtained. The content of Fig. 4C is then subtracted from Fig. 4B. After median filter, most minor impurities and noise are removed, as Fig. 4D shows. Combining Fig. 4D with Fig. 4A, the image of chromosomes is finally obtained, as Fig. 4E shows. The chromosomes can be then retrieved from the image by conventional edge detection algorithms.

### Adhesive chromosome mass segmentation algorithm

Due to variations in the slide-making process, the chromosomes are not uniformly distributed in metaphase cells. As a result, some chromosomes may distribute in masses in metaphase cells. Those adhesive chromosome masses need to be properly classified initially and then segmented to increase the identification rate as much as feasible and assure the correct counting of the number of chromosomes.

**Figure 5:**
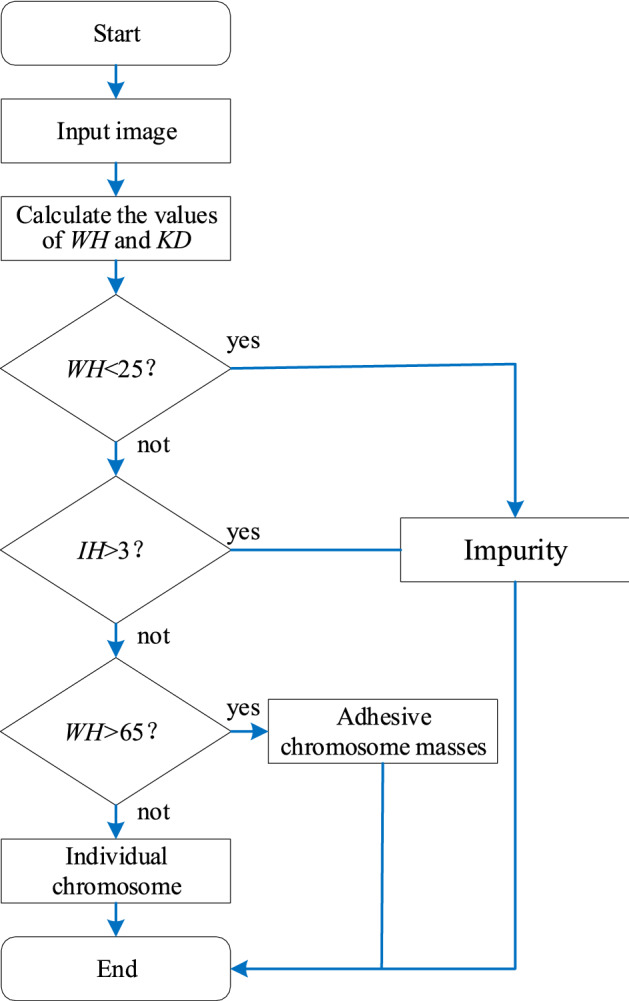
Flow chart of the automatic classification process.

The automatic classification for individual chromosomes and adhesive chromosome masses is primarily based on the morphological characteristics including width (WH) and the number of internal holes (IH). Since the chromosomes in metaphase cells distributes at random angles, WH is defined as the number of pixel points on the short side of the minimum enclosing rectangle for the chromosome. IH is the number of unconnected non-chromosome regions in the chromosome image, which is 0 for general chromosomes, 1 for DICs, 2 for tricentric chromosomes (only found at high radiation doses). The number of IH can increases by a step of 1 for chromosomes with two long arms crossed, so the number of IH is generally no more than 3 for either chromosomes, DICs, or even tricentric chromosomes. The minimum width of the chromosome is 25 pixels and the maximum width of the chromosome is 65 pixels. Therefore, when WH < 25 or IH > 3, the image is judged as an image of impurity; when WH > 65, the image is judged as an image of adhesive chromosome masses; the rest of the images are judged as images of individual chromosome. The automatic classification process is depicted in Fig. 5.

All images obtained from the chromosome extraction algorithm are classified into three categories: individual chromosome images, adhesive chromosome mass images, and impurity images. The individual chromosome images are directly proceeded to the next step, the adherent chromosome masses are processed in automatic segmentation, and the impurity images are discarded outright.

**Figure 6:**
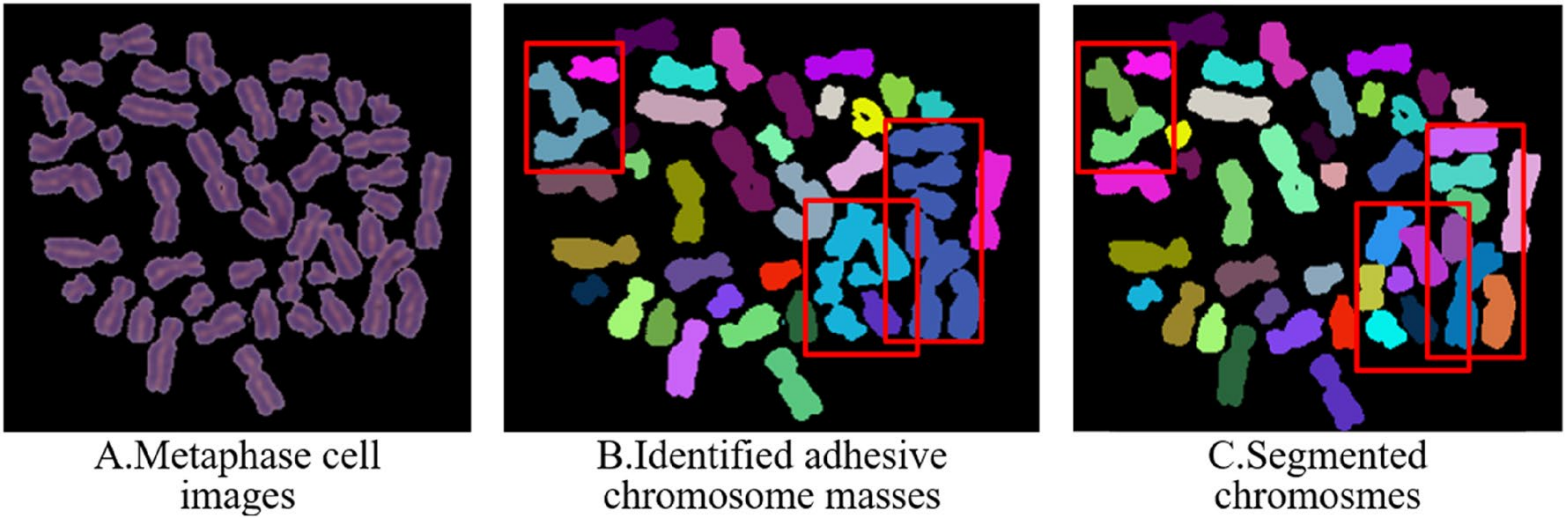
Process of automatic segmentation of adhesive chromosome masses.

The watershed algorithm^32^ is employed to segment adhesive chromosome masses. However, it is still unable to segment chromosome masses with a high degree of crossover. Since the shapes and sizes of various chromosomes vary significantly, the algorithm includes a judgment of the seed point area threshold to filter out erroneous seed points that are too tiny, preventing over-segmentation. The minimum seed point threshold in this method is set at 150 pixels. Figure 6A shows the original metaphase cell images, Fig. 6B displays three groups of adhesive chromosome masses, and Fig. 6C illustrates the automatic segmentation results of these adhesive masses.

### Dicentric chromosome image identification using two-stage CNN

**Figure 7:**
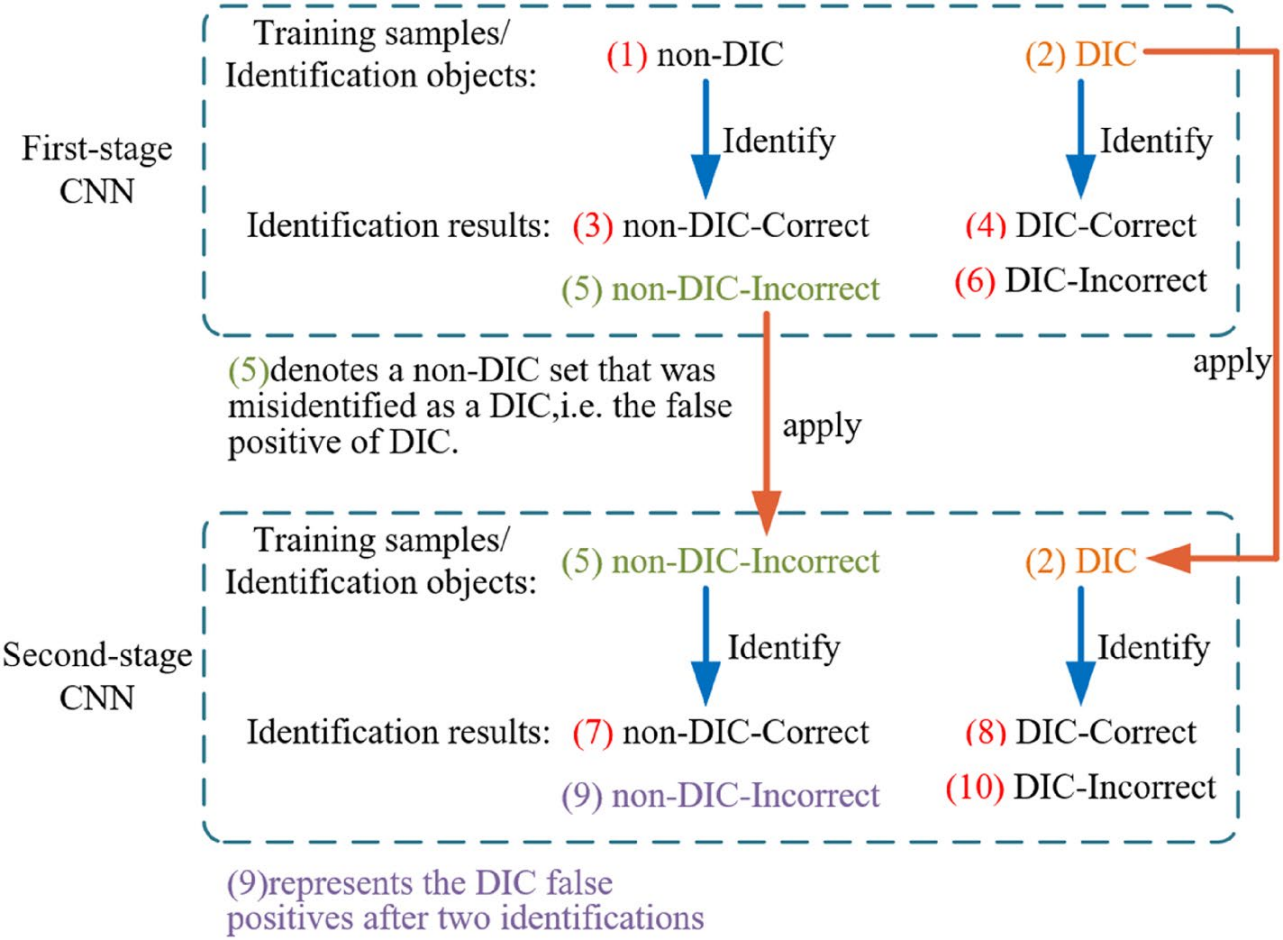
Two-stage convolutional neural network identification model architecture.

After the previous processes, about 710,000 chromosome images are obtained for the 40 sample slides. Five experts manually classify these images for three months and found 13,000 DIC images with a non-DIC to DIC ratio of roughly 70:1. Two-stage CNN is used for automatic identification, as shown in Fig. 7. The first-stage CNN is employed to recognize all the 710,000 images. The chromosome images are divided into (1) 700,000 non-DIC and (2) 13,000 DIC. Due to the identification error of this model, a tiny percentage of the non-DIC images in dataset (1) is misidentified as DIC, resulting in dataset (5), which presents false positive for DIC. Then the second-stage CNN is used for further image identification for datasets (5) and (2). The dataset (9) represents the final false positive for DIC.

According to the current hardware market, there is no graphics card whose memory is large enough to include all the 700,000 non-DIC images in the first-stage CNN. Therefore, in the training process, 100,000 images are randomly chosen from 700,000 non-DIC images to reduce overfitting of the first-stage CNN. All 13,000 DIC images are used for training. The non-DIC and DIC images are randomly assigned to be training set, validation set, and test set with a ratio of 7:2:1.

Before training, the average size of chromosome images is calculated to be 151 × 151 pixels. All the chromosome images are stretchlessly zoomed to 151 × 151 pixels. To improve the stability of the CNN models, image enhancing methods are used to mirror, pan, and add noise to the chromosome images.

**Figure 8:**
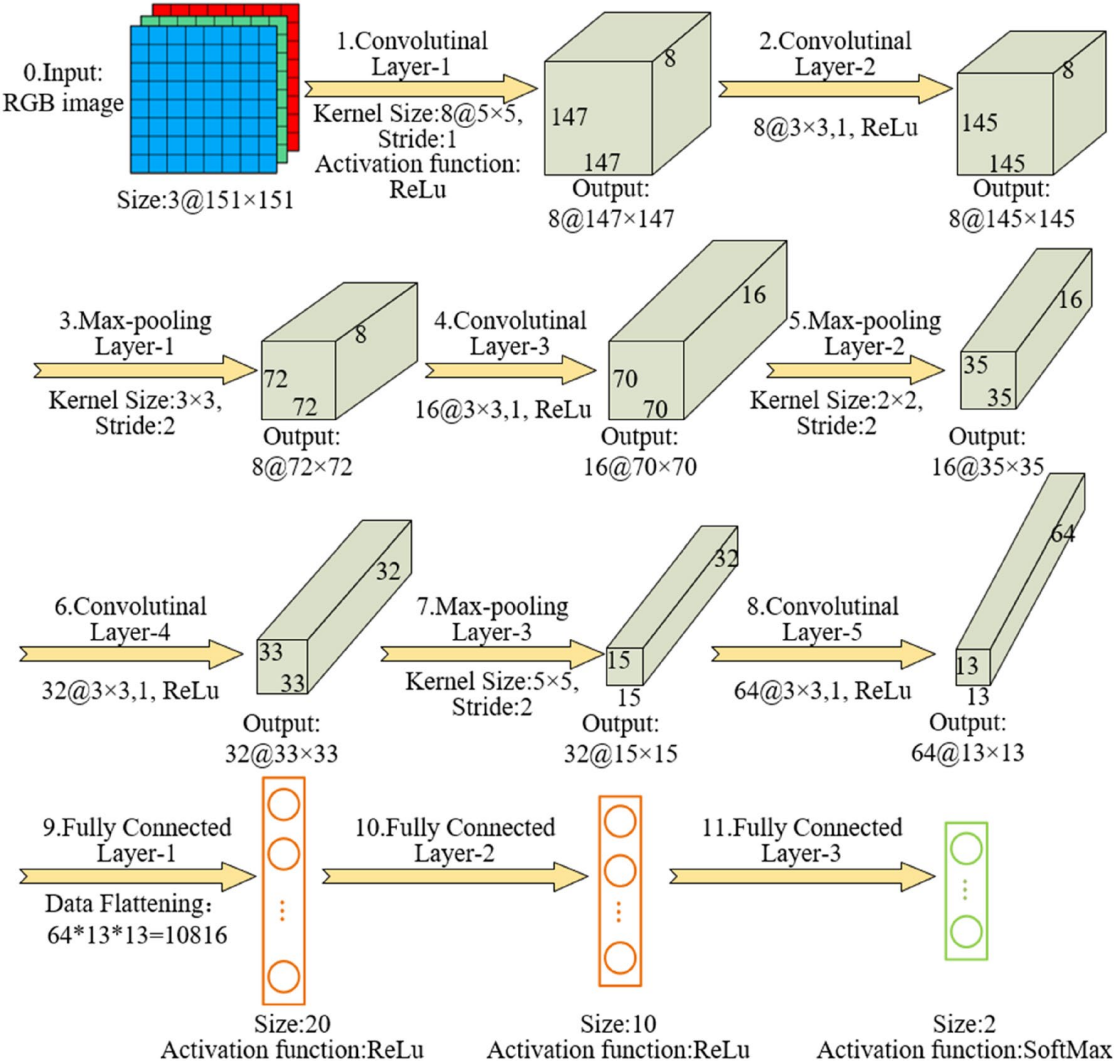
Overview of the first-stage CNN model.

After a few trials, the first-stage CNN model with eight layers is established (5 convolutional layers and 3 fully connected layers), as shown in Fig. 8. Convolutional layer-1 filters the 3 × 151 × 151 input image in RGB mode using 8 filters of 5 × 5; convolutional layer-2 uses 8 filters of 3 × 3; convolutional layer-3, convolutional layer-4, and convolutional layer-5 use 16, 32 and 64 filters of 3 × 3, respectively. Between convolutional layer-2 and convolutional layer-3, a maximum pooling layer with a filter of 3 × 3 is used. Between convolutional layer-3 and convolutional layer-4, a maximum pooling layer with a filter of 2 × 2 is used. Between convolutional layer-4 and convolutional layer-5, a maximum pooling layer with a filter of 5 × 5 is applied.

The fully connected layer-1 has 20 neurons, and fully connected layer-2 has 10 neurons. To introduce the non-linearity relationship between the layers, a rectified linear unit (ReLU) activation function is applied after each convolutional and fully connected layer. A softmax function is deployed at the fully connected layer-3 and outputs an array with the values between 0 (DIC) and 1 (non-DIC). The DIC images of training and validation sets are 3 times oversampled during model training to increase DIC identification accuracy since the quantity of DIC images is limited. The learning rate is set to be 0.001 and reduced by a factor of 10 after half iteration of training. The momentum term is set to be 0.9, the weight decays to 0.0001, the batch size is set to be 256. All parameter settings are standardized except those for the specified configuration. The first-stage CNN model is trained in 50 iterations for nearly 70 h on a NVIDIA GeForce GTX 3090 GPU.

The second-stage CNN also contains 8 layers (5 convolutional layers, 3 fully connected layers). Convolutional layer-1 has 16 filters of 5 × 5; convolutional layer-2 has 24 filters of 3 × 3; convolutional layer-3, convolutional layer-4, and convolutional layer-5 have 40, 40, and 64 filters of 3 × 3, respectively. The fully connected layer-1 has 20 neurons, and fully connected layer-2 has 10 neurons.

To boost the identification accuracy of the second-stage CNN model, the training and validation sets of DIC images are oversampled by 2. To avoid the overfitting problem caused by oversampling, dropout regularizations with coefficients of 0.5 and 0.3 are applied before the fully connected layer-1 and the fully connected layer-2. The other training parameters are the same as those used in the first-stage CNN model.

## Results and discussions

### Identification accuracies of the CNN models

**Figure 9:**
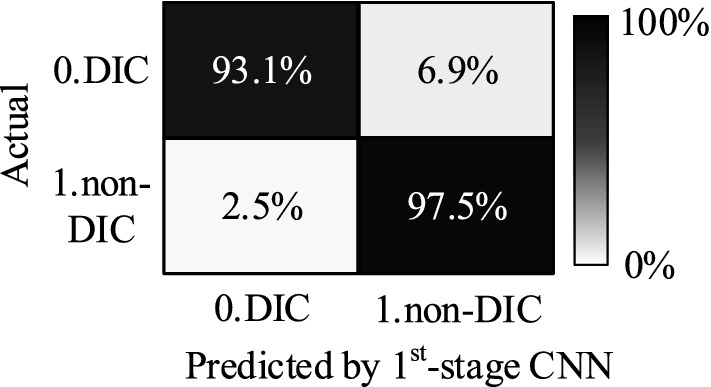
Identification accuracy of the first-stage CNN model.

During the training phase, the first-stage CNN model converges well. Using images from the test set to test the first-stage CNN model, the overall identification accuracy reaches 97% with identification accuracies of 97.5% for Non-DIC images and 93.1% for DIC images, as shown in Fig. 9.

The remaining 600,000 non-DIC images are then identified by the first-stage CNN model. The final test result of 700,000 non-DIC images obtains an identification accuracy of 97.6% where around 17,000 non-DIC images are misclassified as DIC images. As the identification accuracy of all non-DIC images is higher than that of the test set for first-stage CNN model (97.5%), the first-stage CNN model is effectively trained and has strong applicability to identify new chromosome images.

**Figure 10:**
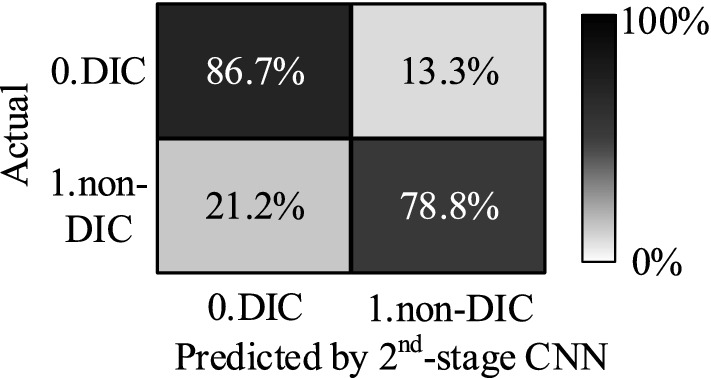
Identification accuracy of the second-stage CNN model.

The second-stage CNN model also converges well during the training phase. Using images from the test set of the second-stage CNN model, the overall identification accuracy reaches 82.8% with identification accuracies of 78.8% for non-DIC images and 86.7% for DIC images, as shown in Fig. 10.

The identification accuracy of the second-stage CNN model is limited by number of training images. In this research, it took nearly three months to manually select and classify nearly 710,000 chromosome images with the help of five experts. Only about 30,000 images could be used for the training of the second-stage CNN model.

### Identification accuracies of the identification method using two-stage CNN

**Figure 11:**
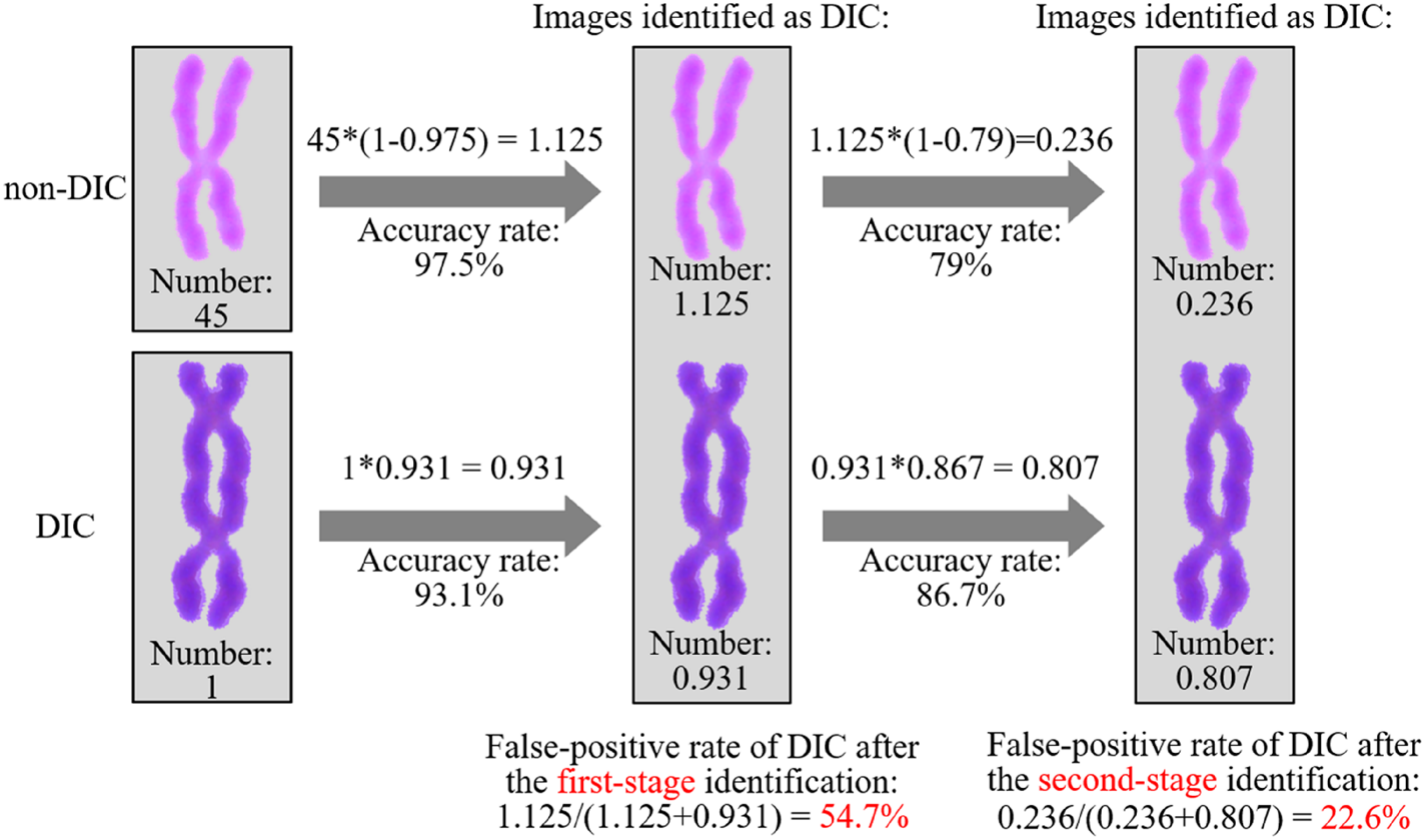
False positive rate of DIC identified by identification method using two-stage CNN.

To analyze the identification performance of novel automatic identification method using two-stage CNN, Fig. 11 presents the false positive rate of DIC identified by identification method using two-stage CNN (assuming the ratio of non-DIC to DIC is 45:1). After the first-stage identification, DIC has a false positive rate of 54.7%. After the second-stage identification, the false positive rate of DIC is decreased to 22.6%. The false positive rate of DIC can be further decreased if the identification accuracy of the second-stage CNN model can be improved.

In practice, the number of non-DIC is substantially larger than the number of DIC, especially in low-dose samples^29,30^. A random selection of 245 metaphase cell images not included in the training process are used. The images are first manually identified by experts for the DICs. Then the images are automatically identified in two modes, the first-stage CNN only and the two-stage CNN. Manual detection finds 11,270 chromosomes in total and 211 DICs among the 245 metaphase cell images. The identification using the first-stage CNN only identifies 505 DICs with 304 misidentified DICs, resulting in a false positive rate of 60.2%. The identification using two-stage CNN finds 223 DICs with 42 misidentified DICs, obtaining a false positive rate of 18.8%. This suggests that, using the novel identification method, the false positive rate of DIC can be decreased at higher non-DIC to DIC ratio, better suiting practical application.

### Identification performances for typical chromosomes

**Figure 12:**
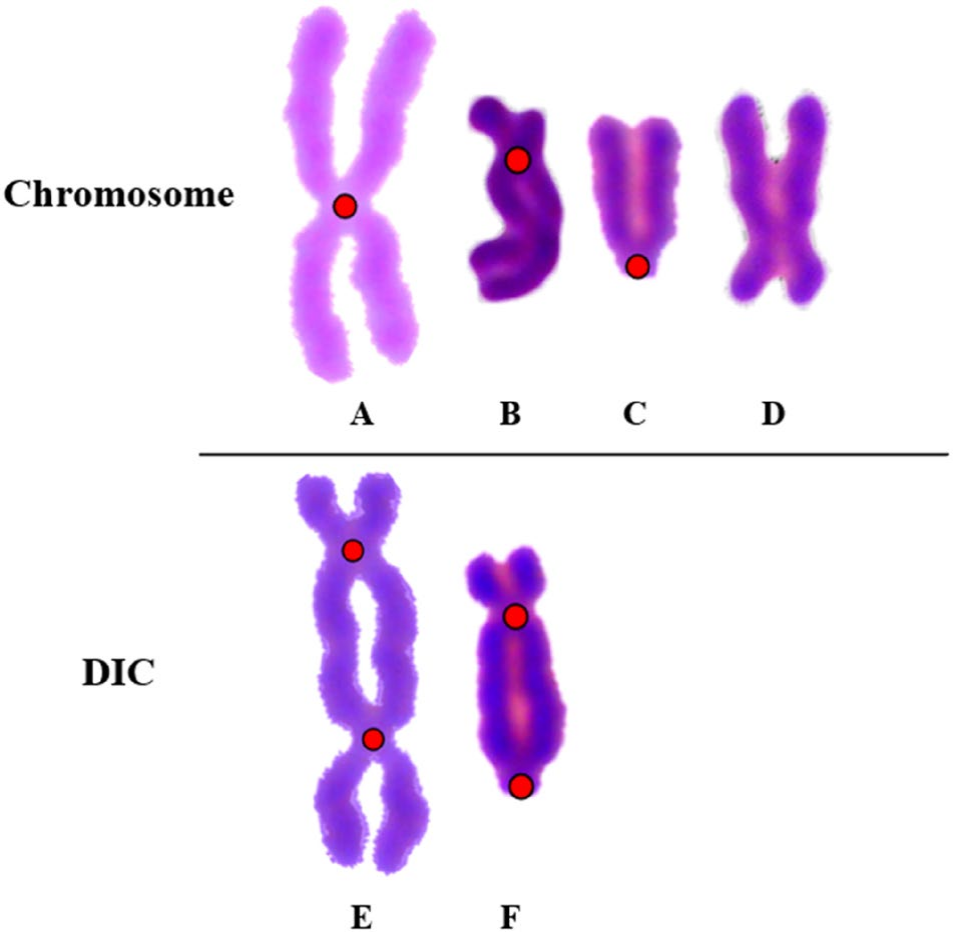
Typical chromosomes and DICs. (**A**) common chromosome, (**B**) chromosome with long arms entangled together, (**C**) chromosome with the centromere at the ends, (**D**) weakly split chromosome, (**E**) common DIC, (**F**) DIC with the centromere at the ends.

Figure 12 shows some typical chromosomes and the DICs. Figure 12A, E illustrate the common chromosomes and DICs. There are some types of chromosomes and DICs hard to identify. Figure 12B displays a chromosome with long arms entangled together, which can be easily mistaken for DIC. Figure 12C, F exhibit chromosomes and DICs with the centromere at the ends and Fig. 12D displays a chromosome weakly split. Conventional identification methods based on morphology and threshold setting are unable to properly detect these complex images, resulting in a high false positive rate.

**Figure 13:**
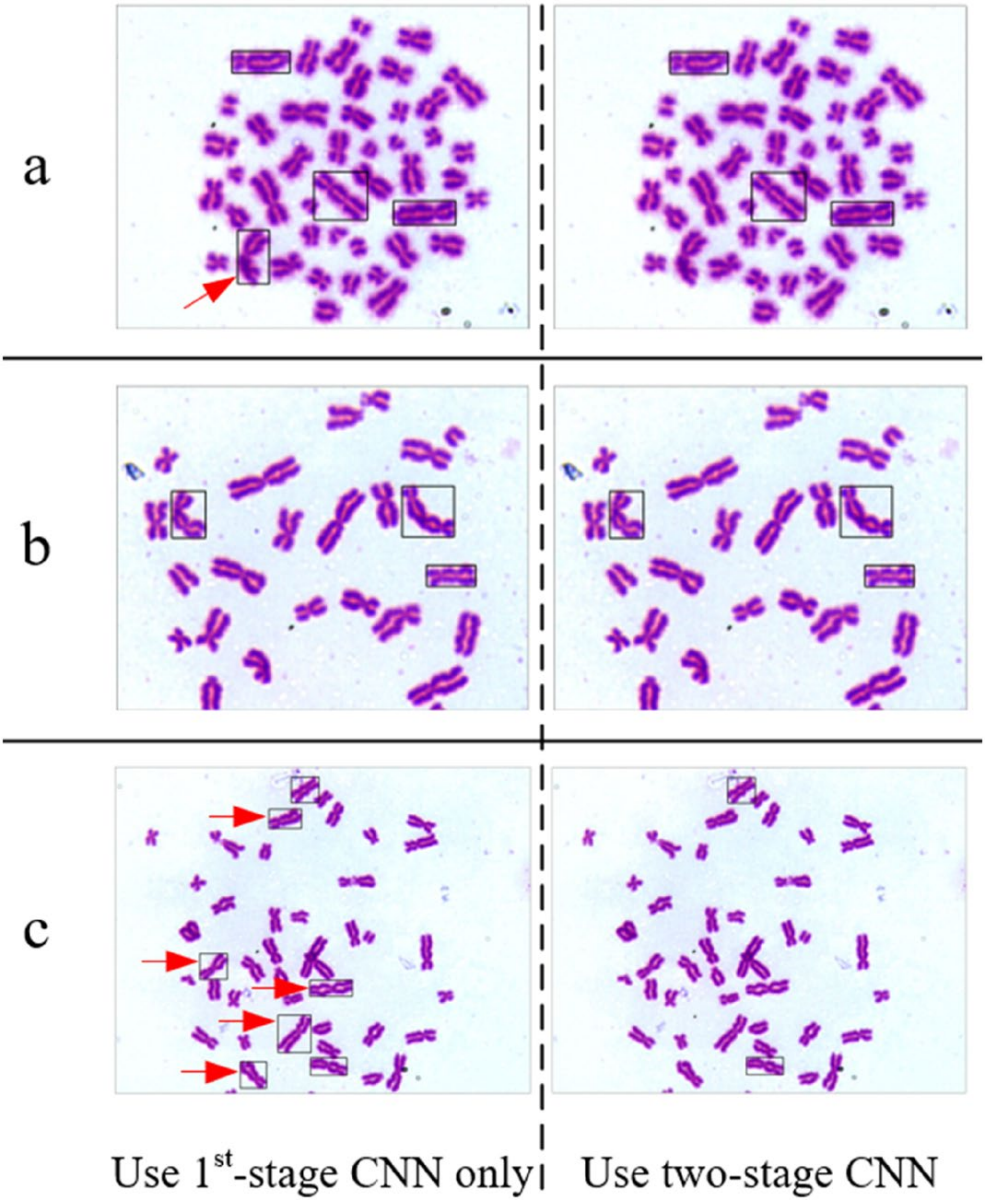
Identification results of two automatic identification modes for three kinds of typical chromosome. (**a**) Image with long arms entangled chromosome, (**b**) image with tricentric chromosome, (**c**) image with DIC.

Figure 13 show the identification results using first-stage CNN only (left) and two-stage CNN (right) for three kinds of typical chromosomes. The misidentifications are pointed out by red arrows. Both identification modes have an overall identification accuracy of at least 95%. Using first-stage CNN only leads to a lower DIC missing rate and using two-stage CNN achieves a lower DIC false positive rate.

According to Fig. 13a, the chromosomes with long arms entangled are easy to be misidentified as DICs. There are 94 long-armed entangled chromosomes in 245 metaphase cell images. The identification using first-stage CNN only misidentifies 51 with an accuracy of 45.7%; the identification using two-stage CNN misidentifies 21 with an accuracy of 77.7%. It suggests on the one hand, the misidentification for chromosomes with long arms entangled is hard to avoid. On the other hand, using the two-stage CNN can achieve a better identification accuracy for long-armed entangled chromosomes than using the first-stage CNN only.

As shown in Fig. 13b, both identification modes can successfully identify tricentric chromosome. However, though the computational weight of one tricentric chromosome is comparable to two DICs in biological dosage calculation, currently, the identification method can only identify the tricentric chromosomes as abnormal but cannot designate them as tricentric chromosomes. Figure 13c shows that, using two-stage CNN can effectively reduce the false positive rate of DIC compared with using first-stage CNN only.

### Error analysis

Though using the two-stage CNN has a false positive rate of DIC much lower than using the first-stage CNN only (42/223 vs 304/505), using the two-stage CNN also has a lower DIC identification rate (181 vs 201). Parameters such as accuracy, sensitivity, specificity, positive predictive value and negative predictive value, are used to further assess the outcomes of the two identification modes. The parameters are defined by Eqs. (1)–(5) and listed in Table 1.

1 $${\text{Accuracy}}:ACC = {{\left( {TP + TN} \right)} \mathord{\left/ {\vphantom {{\left( {TP + TN} \right)} {\left( {TP + FP + TN + FN} \right)}}} \right. \kern-0pt} {\left( {TP + FP + TN + FN} \right)}}$$

2 $${\text{Sensitivity}}\;({\text{true}}\;{\text{positive}}\;{\text{rate}}):TPR = TP/\left( {TP + FN} \right)$$

3 $${\text{Specificity}}\;({\text{true}}\;{\text{negative}}\;{\text{rate}}):TNR = TN/\left( {TN + FP} \right)$$

4 $${\text{Positive}}\;{\text{predictive}}\;{\text{value}}:PPV = TP/\left( {TP + FP} \right)$$

5 $${\text{Negative}}\;{\text{predictive}}\;{\text{value}}:NPV = TN/\left( {TN + FN} \right)$$

where *TP* is the number of ture positive, *FP* is the number of false positive, *FN* is the number of false negative, and *TN* is the number of ture negative.

**Table 1:** Parameters of the two identification methods.

Using first-stage CNN only		Using two-stage CNN	
DIC	Non-DIC	DIC	Non-DIC
*TP* = 201	*FN* = 10	*TP* = 181	*FN* = 30
*FP* = 304	*TN* = 10,755	*FP* = 42	*TN* = 11,017
*ACC*	97.2%	*ACC*	99.4%
*TPR*	95.3%	*TPR*	85.8%
*TNR*	97.3%	*TNR*	99.6%
*PPV*	39.8%	*PPV*	81.2%
*NPV*	99.9%	*NPV*	99.7%

It can be seen that the ACC obtained by the two-stage CNN is slightly higher than that obtained by the first-stage CNN only (99.4% vs 97.2%) while TPR obtained by the two-stage CNN is lower (85.8% vs 95.3%), indicating that using the first-stage CNN only has a lower missing rate. Using two-stage CNN achieves a TNR slightly higher than using the first-stage CNN only (99.6% vs 97.3%), and achieves a PPV significantly higher (81.2% vs 39.8%), revealing that the false-positive rate obtained using two-stage CNN is significantly lower, as well as the positive misdiagnosis rate. Compared with the identification method using first-stage CNN only, the novel automatic identification method using the two-stage CNN tends to be more cautious with a low TPR and high TNR. Compared with manual identification, according to the manual verification, the missing rate of manual identification is around 1%.

The recall (TPR) of the method using first-stage CNN is more than 95% for the test data and using two-stage CNN is more than 85% for the test data, showing a better accuracy compared with previous studies whose TPR are of 50–70%^4,23,29^. The PPV of the method using two-stage CNN is more than 80% for the test data, higher than that in previous paper, 51%^23^. In addition, since DIC only takes a small portion of chromosomes, high false positives will require a significant amount of manual checking time. Considering both accuracy and efficiency, the two -stage CNN shows a good overall performance and can be a useful tool for biological dose assessment. Furthermore, the difference between the identification results of the method using first-stage CNN (high TPR, low PPV) and those of the method using two-stage CNN (low TPR, high PPV), i.e. the chromosomes identified as DIC by using first-stage CNN but non-DIC using two-stage CNN, can be abstracted for further manual check. In this way, TPR can be improved, and the identification quantity can be reduced for dose assessment.

### Time efficiency of the novel method

In literature, conventional identification algorithms passed chromosome cross-sectional pixel information^5^ before iterating skeletonization techniques^12^ to find the medial axis of chromosome using various curve fitting methods^6–8^, handwritten word recognition^9^, or skeleton trimming techniques^10,11^. Finally, the locations of centromeres are determined by the convexity^10,12^ and pixel information^6,7,13^ of the chromosomes. Feature pyramid networks^22^, support vector machines^23^, hierarchical multilayer neural networks^24^, and Siamese networks^25^ are the machine learning algorithms applied to identify and categorize chromosomes. The image filtering neural network^22^ or image morphological features^26,27^ are deployed to filter out poor quality metaphase cell images.

The process of the novel automatic chromosome identification method can be divided into three steps: automatic slide scanning, detecting metaphase cells, and automatic metaphase cell image analysis. Since the distribution of metaphase cells on slides and the dispersion of chromosomes in metaphase cells vary, the time to detect the same number of metaphase cells on slides varies dramatically. For most handcrafted slides, only 10–15% of the slide area needs to be scanned and around 100 metaphase cell images would be obtained. The automatic slide scanning step takes approximately 5–7 min, and the entire identification process takes around 30 min.

Compared with previous works, since this novel method employs CNN models for image identification, it eliminates the need for tedious image identification operations like manual feature extraction, dimensionality reduction, and feature ranking. The CNN can intelligently alter network settings to get the best classification performance, as well as extract image features automatically by building image features after each convolutional layer and determining the ideal proportion of image features. During the identification process, this novel identification method requires no manual classification of chromosomes/DICs from impurities/acentric fragments in metaphase cells before DIC identification.

According to statistics, the novel automatic identification method takes around 1500 s to analyze 100 images of metaphase cells while manual identification takes about 300 s for per metaphase cell image. This novel automatic identification method is about 20 times more efficient than manual identification which significantly enhances the speed of the biological dose assessment.

## Conclusion

A high-precision automatic identification method for dicentric chromosome image using two-stage CNN is proposed. This novel method achieves a low DIC false positive rate and high image processing speed for a random selection of 245 unused metaphase cell images. The following conclusions may be drawn:

1. The method using first-stage CNN only yields a chromosome identification accuracy of 97.2%, sensitivity of 95.3%, and specificity of 97.3%. The two-stage CNN method, combining two CNN models, has an identification accuracy of 99.4%, a sensitivity of 85.8%, and a specificity of 99.6%.
2. High sensitivity (TPR) and high positive predictive value (PPV) cannot be attained at the same time, the method using first-stage CNN only has a lower miss rate (4.7% vs 14.2%) while the method using the two-stage CNN has a lower false positive rate (18.8% vs 60.2%).
3. Using the method of two-stage CNN, at a target rate of 1:53 in 245 metaphase cell images, the false positive rate of DIC is 18.8%. This novel method can provide a valuable reference for other image identification situations with small target rates.

## Data Availability

The datasets used and/or analysed during the current study available from the corresponding author on reasonable request.
